# Genetic characterization of Stargardt clinical phenotype in South Indian patients using sanger and targeted sequencing

**DOI:** 10.1186/s40662-019-0168-8

**Published:** 2020-01-09

**Authors:** Rajendran Kadarkarai Raj, Pankaja Dhoble, Rupa Anjanamurthy, Prakash Chermakani, Manojkumar Kumaran, Bharanidharan Devarajan, Periasamy Sundaresan

**Affiliations:** 1 Department of Genetics, Aravind Medical Research Foundation-Madurai, No.1 Anna Nagar, Madurai, Tamil Nadu 625 020 India; 2 Retina Consultant, Department of Vitreo Retinal services, Aravind Eye Hospital-Pondicherry, Puducherry, India; 3 Department of Paediatrics and Adult strabismus, Aravind Eye Hospital-Madurai, Madurai, Tamil Nadu India; 4 Department of Bioinformatics, Aravind Medical Research Foundation-Madurai, Madurai, Tamil Nadu India

**Keywords:** Stargardt, *ABCA4*, Macular degeneration, Yellow white flecks, Mutation detection, South Indian population

## Abstract

**Background:**

Stargardt disease 1 (STGD1; MIM 248200) is a monogenic form of autosomal recessive genetic disease caused by mutation in *ABCA4*. This gene has a major role in hydrolyzing N-retinylidene-phosphatidylethanolamine to all-trans-retinal and phosphatidylethanolamine. The purpose of this study is to identify the frequency of putative disease-causing mutations associated with Stargardt disease in a South Indian population.

**Methods:**

A total of 28 clinically diagnosed Stargardt-like phenotype patients were recruited from south India. Ophthalmic examination of all patients was carefully carried out by a retina specialist based on the stages of fundus imaging and ERG grouping. Genetic analysis of *ABCA4* was performed for all patients using Sanger sequencing and clinical exome sequencing.

**Results:**

This study identified disease-causing mutations in *ABCA4* in 75% (21/28) of patients, 7% (2/28) exhibited benign variants and 18% (5/28) were negative for the disease-causing mutation.

**Conclusion:**

This is the first study describing the genetic association of *ABCA4* disease-causing mutation in South Indian Stargardt 1 patients (STGD1). Our findings highlighted the presence of two novel missense mutations and an (in/del, single base pair deletion & splice variant) in *ABCA4*. However, genetic heterogeneity in *ABCA4* mutants requires a larger sample size to establish a true correlation with clinical phenotype.

## Background

Stargardt disease (STGD) is a monogenic form of juvenile macular degeneration, which was first described by Karl Stargardt in 1909 [1, 2]. The globally estimated prevalence rate is 1 in 8000–10,000. It is characterized by early central vision loss, progressive degeneration of the macula that is associated with loss of photoreceptors leading to irreversible vision loss [3, 4]. Yet, another important unique characteristic clinical feature is the presence of distinct yellow-white flecks around the macula and mid-periphery of the retina [5]. The disease symptoms typically develop as early as in the first or second decade of life. Genes associated with degenerative macular dystrophies are highly expressed in photoreceptor cells playing a crucial role in phototransduction, visual cycle, photoreceptor structure and small molecule transport [6]. STGD1 is one of the most common autosomal recessive inherited retinal disorders caused by a mutation in the ATP Binding Cassette Subfamily A Member 4 (*ABCA4*) gene, whereas, mutations in elongation of very-long-chain fatty acids 4 (*ELOVL4*), prominin 1 (*PROM1*) genes are responsible for the STGD3 and STGD4 phenotype, respectively [7, 8].

The *ABCA4* gene located in chromosome 1p22.1 contains 50 exons that codes for a membrane bound glycoprotein that is ubiquitous and localized to the rim of the rod and cone outer discs membrane [9]. In addition, it is actively involved in the transport of retinoid substrate from photoreceptor to RPE [10]. Indeed, mutation in *ABCA4* affects the retinoid transport activity, which subsequently affects the clearance of all-trans-N-ret-PE in the rod disc membrane. Consequently, the waste product, all-trans-N-ret-PE, reacts with all-trans-retinal forming dihydropyridinium compounds, which undergo auto-oxidation and thereby generate phosphatidyl-pyridinium bisretinoid A2PE in photoreceptors. So far, more than 1000 mutations have been reported in *ABCA4* across different cohorts leading to STGD1 and other retinal disorders like autosomal recessive cone-rod dystrophies, age macular degeneration and retinitis pigmentosa [11]. To our knowledge, only one study reported the clinical and genetic correlation of STGD1 disease in five families belonging to of Indian origin [12].

The current study utilized a combinatorial approach including conventional Sanger sequencing and Targeted exome sequencing (TES) to determine the frequency of putative disease-causing variants associated with Stargardt disease in a South Indian population.

## Methods

### Study samples and clinical assessment

We recruited 28 clinically diagnosed Stargardt disease-like phenotype patients from two territories of Aravind Eye hospital-Madurai & Pondicherry, India, between 1998 and 2007 and 2018–2019. All the study participants are of South Indian origin (Tamil Nadu, Pondicherry, Kerala, Andhra Pradesh and Karnataka). The ophthalmic features were carefully examined in both eyes by a retina specialist. The examination included patient’s age, disease onset, best corrected visual acuity (BCVA-Snellen acuity chart), slit lamp biomicroscopy, color fundus photography (TRC-50IA Retinal Fundus Camera) (Topcon, Inc., Tokyo, Japan), Spectral-domain optical coherence tomography (SD-OCT), Autofluorescence (AF) images using Spectralis with viewing module version 5.1.2.0, Clinical full-field electroretinography (ERG) through Diagnosys Color Dome (Diagnosys LLC, Lowell, MA) based on the standards of the International Society for Clinical Electrophysiology of Vision.

The written informed consent form was received from all probands or parents/legal guardians in cases of minor subjects after explaining the genetic study. A complete clinical and familial pedigree was collected from each proband. This study was approved by the Institutional Ethics Review Board, Aravind Eye Hospital, Madurai, Tamil Nadu, India. The study adhered to the tenets of the declaration of Helsinki.

### Mutation screening

Two methods were adopted to identify the frequency of *ABCA4* mutations in STGD1 patients. Sanger sequencing was performed for 24 samples and the remaining 4 cases were analyzed by a clinical exome sequencing method.

### Polymerase chain reaction (PCR) for *ABCA4*

Five milliliters of peripheral blood were collected from all study subjects using an EDTA-vacutainer. Genomic DNA was extracted using modified salting out precipitation method [13]. Primers were designed for all fifty exons of *ABCA4* (NG_009073.1) with the respective exon - intron boundaries using Primer3 and Primer BLAST software. Fifty nanograms of genomic DNA template was used for all *ABCA4* specific exon amplification with 1 unit of Taq DNA polymerase (Sigma), 50 μM dNTPs (Sigma), 5 pm/μl of forward/reverse primers and standard 1X PCR buffer (Sigma). Gradient PCR was established to optimize the annealing temperature (54 °C - 66 °C) of primers for all 50 exons of *ABCA4*. The PCR amplicon was purified using Exonuclease I-Shrimp alkaline phosphatase reagent (Exo-SAP; Affymetrix, Santa Clara, CA, USA). Further, the samples were sequenced using Big Dye Terminator ready reaction mix using the ABI-3500 genetic analyzer (Applied Biosystems, Foster city, CA).

### Sanger sequencing

Direct sequencing was performed through di-deoxy nucleotide chain termination method to detect the potential variants associated with disease. Sequencing results were viewed in Finch TV and compared with the cDNA sequence of *ABCA4* (NM_0 00350.3). The zygosity status of the variants across the exons (homozygous, heterozygous and compound heterozygous) was also identified through chromatogram.

### Mutation evaluation

All the variants obtained from Sanger sequencing were predicted for its pathogenicity using the following in silico tools: The Sorting Intolerant from Tolerant (SIFT) [14], PolyPhen-2 [15], Human Splicing Finder (HSF3.0) [16], Mutation taster [17] and MetaLR [18].

### Targeted exome sequencing (TES)

Targeted exome sequencing was performed for 4 study participants. Cev3 clinical-exome panel was used for library preparation and probe capture. Using Illumina HiSeq X ten platform, 6800 clinically relevant genes were captured with the preconstructed library to generate 150 bp paired-end reads at 100X sequencing depth. Post-sequencing data processing and variants filtration was performed using in-house UNIX scripts [19]. The quality of the raw data in FASTQ file was checked and the bad reads were removed using Fast QC (version 0.11.5). The read alignment was done using BWA-MEM aligner (version 0.7.12-r1039) (23). The PCR-duplicate reads from the aligned reads were removed using Picard mark duplicate (version 2.18.24). The aligned reads were compared with hg19 reference version from UCSC genome browser. Further, single nucleotide polymorphisms (SNPs), point mutations and short indels were prioritized using Haplotype Caller module in GATK (version 4.0). These variants were finally annotated using ANNOVAR [20] to predict whether the mutation was silent, mis-sense or nonsense.

### Variants prioritization

The variants obtained from ANNOVAR file were prioritized by applying a stringent filter with minor allele frequency (MAF) less than or equal to 0.1% in 1000genome, ESP, ExAC and gnomAD. Only the non-synonymous coding or splice-site variants with the conservation score > 2.5 (GERP score) and CADD score greater than 10 were selected. To predict the deleteriousness, the variants were further analyzed using in silico tools like Polyphen2, SIFT, Mutation Taster, FATHMM and LRT. Finally, the filtered variants were ranked out by their association with Stargardt disease using VarElect software [21].

### Conservation analysis and effect of missense mutations in protein stability

Multiple sequence alignment was carried out using the Clustal Omega online tool. The structure of the ABCA4 domain was predicted through I-TASSER online software (http://zhanglab.ccmb.med.umich.edu/I-TASSER/). The predicted structure was evaluated by mutation cutoff scanning matrix (mCSM), site-directed mutator SDM and DUET server, which calculated the stability difference score between the wild and mutant type protein [22].

## Results

### Disease-causing mutations identified by sanger sequencing and TES

**Fig. 1:**
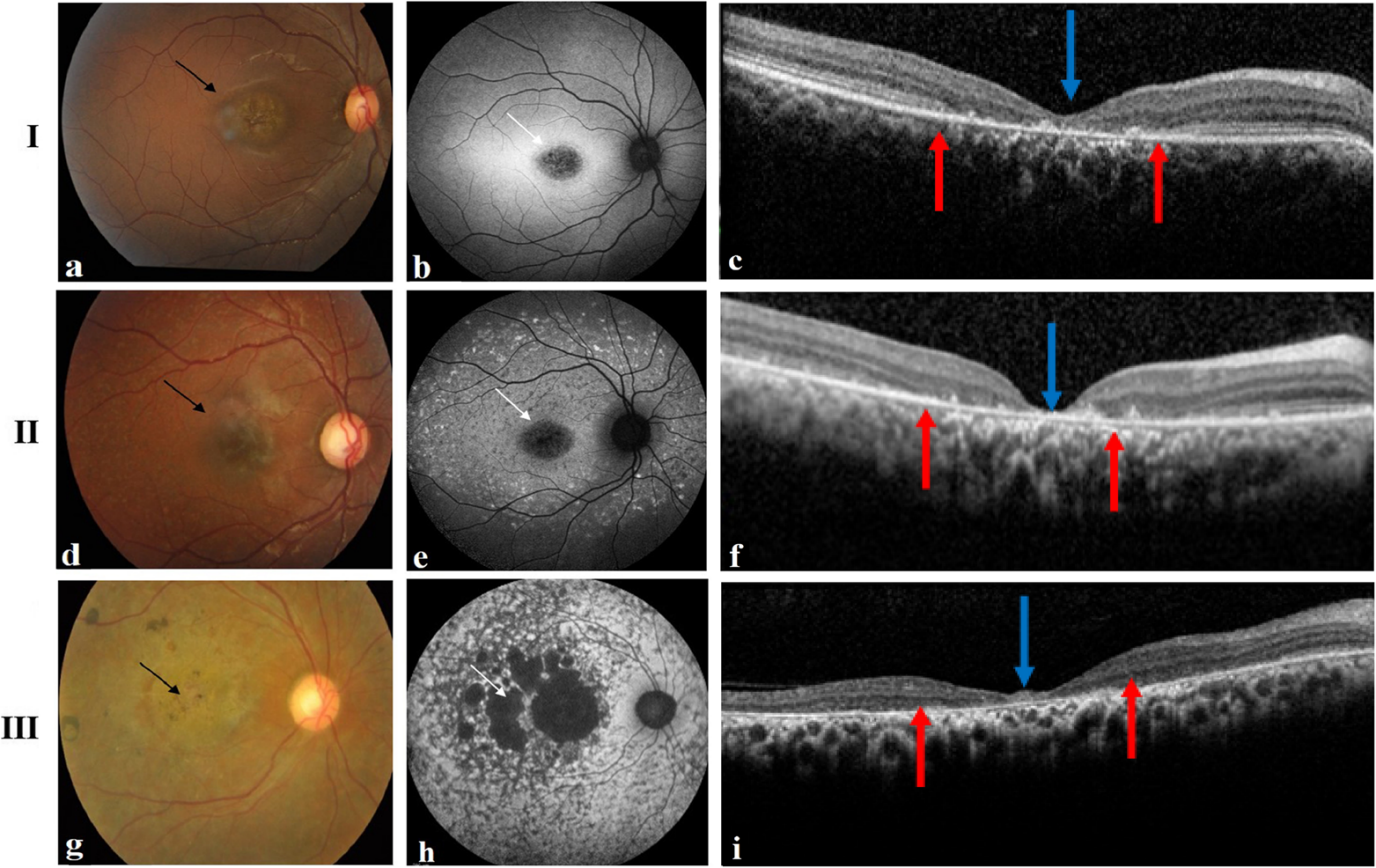
Representative Fundus, Autofluorescence and SD-OCT images of STGD1 patients. The panels (I, II, III) represent the images of Fundus, AF and SD-OCT of case IDs: 27, 24, and 25, respectively. Panel I: **a** Fundus photos of the patient’s right eye. The black arrow indicates the atrophic lesions at the macula. **b** Corresponding fundus autofluorescence image in the central area represents hypoautofluorescence (white arrow), with surrounding flecks showing hyper and hypoautofluorescence. **c** SD-OCT image indicates foveal thinning (blue arrow) and the loss of outer retinal layers (red arrows). Panel II: **d** Fundus photos of the patient’s right eye denoting the central atrophic macula (black arrow). **e** AF shows the corresponding area of central hypoautofluorescence (white arrow) and hyperautofluorescence of flecks. **f** SD-OCT image indicates the foveal thinning (blue arrow) and the loss of photoreceptors centrally (red arrows). Panel III: **g** Fundus photos of the patient’s right eye. The image represents the central atrophic macula (black arrows) as well as the extensive loss of choriocapillaries and RPE atrophy throughout the macula and beyond. **h** AF shows large areas of hypoautofluorescence (white arrows). **i** Central foveal thinning (blue arrow) and loss of photoreceptors was evident upon SD-OCT imaging (red arrows).

In the present study, 28 patients with clinically Stargardt disease-like phenotype were recruited. All the affected probands presented with complaints of defective vision or central vision loss in both eyes, of which the ophthalmic evaluation was carefully carried out only in 11 patients who were taken forward for further phenotype classification (Table 1) and segregation analysis (Additional file 1: Table S2). The disease progression of STGD1 based on fundus imaging (Fishman’s classification) [23] and ERG grouping [24] (Fig. 1) was keenly categorized by our clinicians. Of the total 11 probands, 27% were diagnosed with stage-1 disease, 36% were categorized as stage-2, 27 and 9% had stages 3 and 4, respectively. Use of non-invasive retinal imaging, especially AF, enabled improved visualization of fundus changes that would have otherwise been challenging to visualize fundoscopically. According to full field ERG, 27% of probands belonged to group-1 as well as group-2 and 45% were categorized as group-3. SD-OCT findings indicated the following phenotypes such as RPE thinning, IS-OS loss/disruption, outer retinal thinning and macular atrophy. These phenotypes were commonly observed in all probands. Case ID 22 showed a Bulls eye maculopathy-like fundus, but OCT was similar to other phenotypes.

**Table 1:** Clinical Phenotypic features of south Indian STGD1 patients

ID	Age/Sex	Age of onset	BCVA in BE	Fundus	Auto Fluorescence	SD-OCT	ERG
17	16/M	9	20/80	Foveal atrophy few flecks in macula	Hypo AF and hyper AF flecks observed in nasal to optic disk	IS/OS loss	Normal
18	8/M	9	20/80	Macular atrophy	Central hypo –AF, few hyper-AF flecks surrounding	IS/OS loss	Cone dysfunction
19	17/F	6	< 20/200	Mild temporal pallor; flecks in macula and posterior pole; RPE and choroidal atrophy, extensive loss of choriocapillaries throughout fundus	Central hypo-AF, hyper and hypo AF flecks all over fundus	IS/OS loss	Cone-rod dysfunction
20	12/M	10	20/80	Macular atrophy with choriocapillary atrophy, few yellow flecks in macula	Central hypo-AF, hyper-AF Flecks, surrounding atrophy, hyper and hypo-AF flecks all over the fundus	IS/OS loss	Cone-rod dysfunction
21	14/M	14	20/50	Foveal atrophy	Central hypo-AF surrounded by hyper-AF flecks	IS/OS loss	Cone dysfunction
22	7/M	6	20/80	Homo Macular atrophy, Bulls eye macula	Central hypo-AF, surrounding ill-defined hyper-AF ring hyper-AF flecks around post pole, extending nasally anterior to vascular arcades	IS/OS loss	Cone-rod dysfunction
24	10/F	7	20/80	Macular atrophy and flecks	Central hypo-AF surrounded by hyper-AF Flecks	IS/OS loss	Normal
25	30/F	7	20/120	Macular atrophy, extensive loss of choriocapillaries and RPE atrophy	Hypo-AF	IS/OS loss	Cone-rod dysfunction
26	10/M	9	20/200	Macular atrophy and flecks	Hypo-AF surrounded by hyper and hypo-AF flecks	IS/OS loss	Cone-rod dysfunction
27	16/F	14	20/80	Macular atrophy	Central area of hypo-AF with surrounding ring of hyper-AF	IS/OS loss	Normal
28	16/M	15	20/50	Foveal atrophy with yellow flecks in post pole, extending anterior to vascular arcades	Central hypo-AF; few flecks anterior to vascular arcade	IS/OS loss	Normal

*BCVA* = best corrected visual acuity; *BE* = both eyes; *AF* = autofluorescence; *SD-OCT* = spectral domain optical coherence tomography; *ERG* = electroretinography; *IS/OS* = inner segment / outer segment layer

**Fig. 2:**
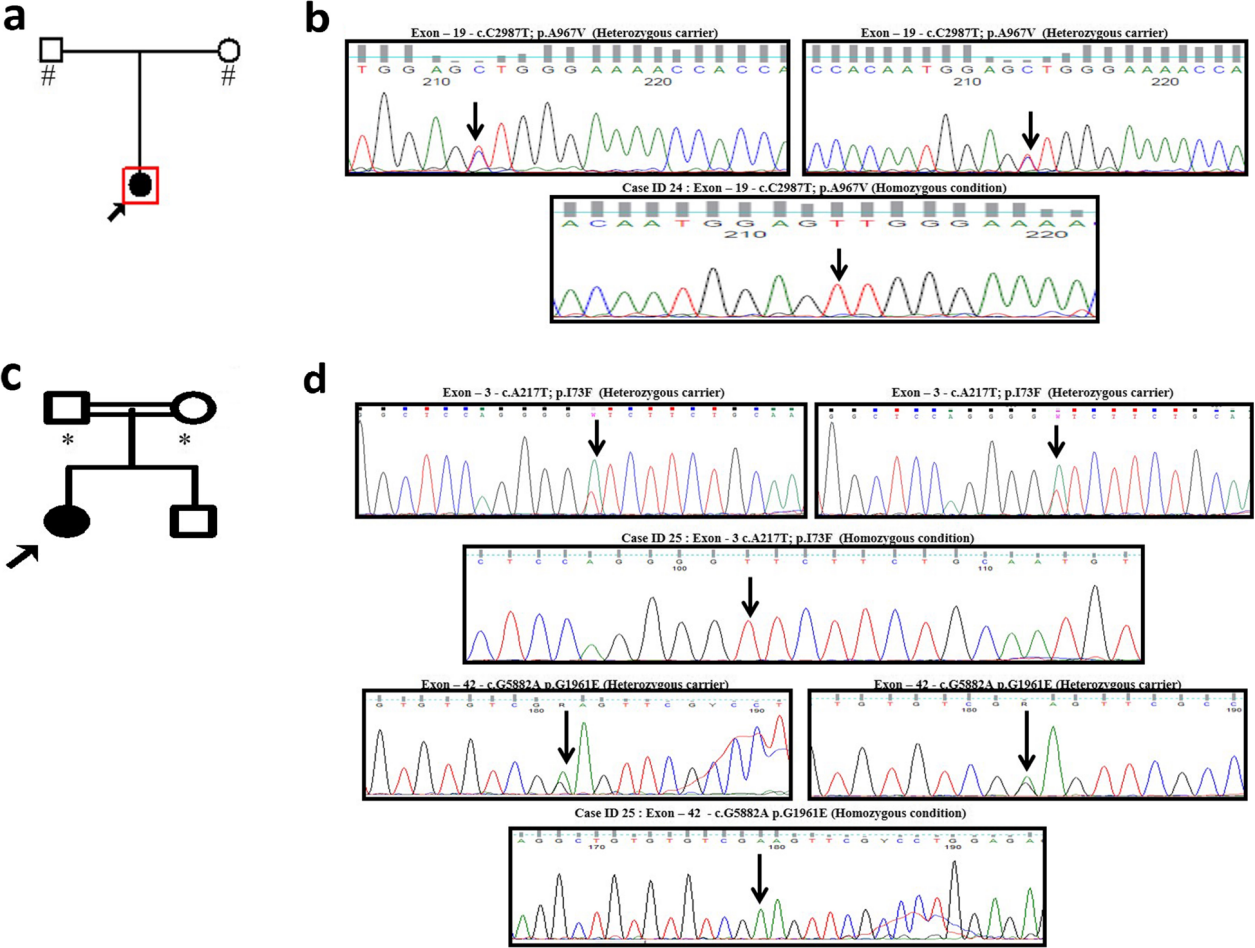
Segregation analysis of *ABCA4* for Case IDs 24 and 25. **a** Case ID: 24 shows no consanguinity between parents. The shaded symbol indicates the affected member, and the open symbols indicate the unaffected members. # - samples were included for genetic analysis. **b** Sanger results demonstrated that the proband harbored a homozygous mutation (c.C2987T) in ABCA4 exon-19. Segregation analysis represented that both the parents were carrier for c.C2987T variant. **c** Case ID: 25 shows consanguinity between parents. * - samples were included for genetic analysis. **d** Targeted exome sequencing results revealed two missense variants in *ABCA4* exon 3 and 42. The disease-causing variants were further validated through Sanger-based method for proband and segregation analysis was performed for parents.

The study adopted two methods. Primarily, 24 samples were screened through Sanger sequencing (Fig. 2a-b) and to further elucidate the disease-associated variants in other STGD-related genes such as *ELOVL4, CNGB3* and *PROM1*, targeted exome sequencing was carried out. TES disclosed the presence of disease-causing mutation only in *ABCA4* (Fig. 2c-d) whereas non-pathogenic variants were observed in clinically relevant STGD genes such as *ELOVL4, CNGB3* and *PROM1* (Additional file 1: Table S1). These results narrowed down our search exclusively to *ABCA4* of the affected STGD patients. Overall, 126 variants in both exonic and intronic boundaries of 29 exons were observed. No variants were detected in any of the other 21 exons. Out of 28 samples, 21 patients showed the presence of disease-causing variants in *ABCA4* exons (Table 2, [6, 25–34]), whereas 2 individuals exhibited benign variants across the *ABCA4* exons, and the remaining 5 samples were negative for *ABCA4* in STGD1 patients. Further, the variants were segregated into homozygous (67%), heterozygous (19%) and compound heterozygous (14%) based on zygosity. Overall, 10 missense mutations that included 2 novel missense mutations, 4 nonsense mutations, a novel in/del, deletion and splice mutation were identified in *ABCA4*.

**Table 2:** List of identified pathogenic mutations across *ABCA4* in STGD1 patients. Genetic analysis of 28 unrelated probands identified *ABCA4* mutation genomic position, nucleotide change and zygosity. Representative symbols show the repository servers used to identify the global allele frequency of variants in healthy control population: ^**&**^ The Exome Aggregation Consortium (ExAC); ^**#**^ Trans-Omics for Precision Medicine (TOPMed) Program; ^*^ The Genome Aggregation Database (gnomAD). Mutations observed in different location of ABCA4 membrane: Transmembrane domain-1 (TMD-1); Transmembrane domain-2 (TMD-2); Extracellular domain-1 (ECD-1); Extracellular domain-2 (ECD-2); Nucleotide binding domain-1 (NBD-1); Nucleotide binding domain-2 (NBD-2)

ID	Exon/Intron	c.DNA change	Amino acid change	Variant Class	Zygosity	Method	MAF	dbSNP	SIFT	PolyPhen	MetaLR	MT	Domain	Reference
2	30	c.C4506A	C1502Ter	Stop Codon	Homozygous	Sanger	0.00001653^**&**^	rs61750149	**–**	**–**	**–**	DC	ECD2	[25]
4	14	c.C1995A	Y665Ter	Stop Codon	Homozygous	Sanger	0.000008536^**&**^	rs757302286	**–**	**–**	**–**	DC	TMD1	[26]
8	13	c.G1819A	G607R	Missense	Homozygous	Sanger	0.00002502^**&**^	rs61749412	D(0)	PD (1)	D(0.933)	DC	ECD1	[27]
9	9	c.G1188A	G396C	Missense	Homozygous	Sanger	N/A	rs866219294	D(0)	PD(0.995)	D (0.804)	DC	ECD1	This study
10	12	c.G1556 T	C519F	Missense	Homozygous	Sanger	–	Novel	D(0)	PD(0.999)	–	DC	ECD1	This study
11	35	c.T4956G	Y1652Ter	Stop Codon	Homozygous	Sanger	N/A	rs61750561	–	–	–	DC	ECD2	[28]
13	42	c.G5882A	G1961E	Missense	Homozygous	Sanger	0.005054^**&**^	rs1800553	D(0)	PD(0.998)	D(0.7)	DC	TMD2	[29]
17	48	c.C6658T	Q2220Ter	Stop Codon	Homozygous	Sanger	0.00000828^**&**^	rs61753046	–	–	–	DC	NBD2	[30]
18	48	c.C6658T	Q2220Ter	Stop Codon	Homozygous	Sanger	0.00000828^**&**^	rs61753046	**–**	**–**	**–**	DC	NBD2	[30]
22	44	c.A6095G	H2032R	Missense	Homozygous	Sanger	0.00000796^**#**^	rs1242866408	D (0)	PD (1)	D (0.924)	DC	NBD2	[31]
24	19	c.C2900T	A967V	Missense	Homozygous	Sanger	0.000003979*	rs1291080436	D (0)	PD (0.99)	D (0.977)	DC	NBD1	This study
25	3,42	c.A217T/c.G5882A	I73F, G1961E	Missense/Missense	Homozygous	ES	0.005054	Novel/rs1800553	D(0)/ D(0)	D(0.92)/PD(0.998)	D(0.983)/D(0.7)	DC	ECD1/NBD2	This study/[29]
26	19	c.C2912A	T971A	Missense	Homozygous	ES	0.000003980^*^	rs61749450	D (0)	PD(0.999)	D (0.989)	DC	NBD1	[6]
27	22	c.G3323A	A1108H	Missense	Homozygous	ES	0.00001649^**&**^	rs61750121	–	–	–	DC	NBD1	[32]
28	19	c.C2912A	T971A	Missense	Homozygous	ES	0.0000039804	rs61749450	D(0)	PD(0.999)	D (0.989)	DC	NBD1	[6]
19	14,19,42	c.C1995A**/**c.C2912A**/**c.G5882A	Y665Ter/T971A/G1961E	Stop codon**/**Missense	Compound Heterozygous	Sanger	0.000008536**/**0.000003980/0.005054/	rs757302286/rs61749450/rs1800553	−/D(0)	PD(0.998)	−/D(0.7)	DC	TMD1**/**NBD1**/**TMD2	[6, 29, 33]
20	46,48	c.6355DelC**/**c.C6658T	Q2220Ter	Del**/**Missense	Compound Heterozygous	Sanger	0.00000828	rs61753046	−/−	−/−	−/−	DC	NBD2/NBD2	This study/[30]
21	26,33	c.C3830T**/**(c.4774-2A > G)	T1277 M	Missense**/**Splice variant	Compound Heterozygous	Sanger	0.0003133^**&**^	rs374565343**/**Novel	D(0.03)	PD(0.835)	D (0.61)	DC	NBD1/ECD2	This study/[30]
5	14	c.C1995A	Y665Ter	Stop Codon	Heterozygous	Sanger	0.000008536	rs757302286	**–**	**–**	**–**	DC	TMD1	[26]
6	26	c.C3830T	T1277 M	Missense	Heterozygous	Sanger	0.0003133	rs374565343	D(0.03)	PD(0.835)	D (0.61)	DC	NBD1	[34]
14	40	c.5710delCAATGinsA	**–**	Ins/Del	Heterozygous	Sanger	–	Novel	**–**	**–**	**–**	DC	NBD2	This study

*IVR* = intronic variant region; *ES* = exome sequencing; *MAF* = minor allele frequency; *D* = deleterious; *PD* = possible or probably damaging; *DC* = disease-causing; *N/A* = not available; *dbSNP* = database of single nucleotide polymorphisms; *SIFT* = The Sorting Intolerant from Tolerant; *MT* = mutation taster

### Modeling of ABCA4-ECD1 domain and predication of protein stability for novel missense variant

**Fig. 3:**
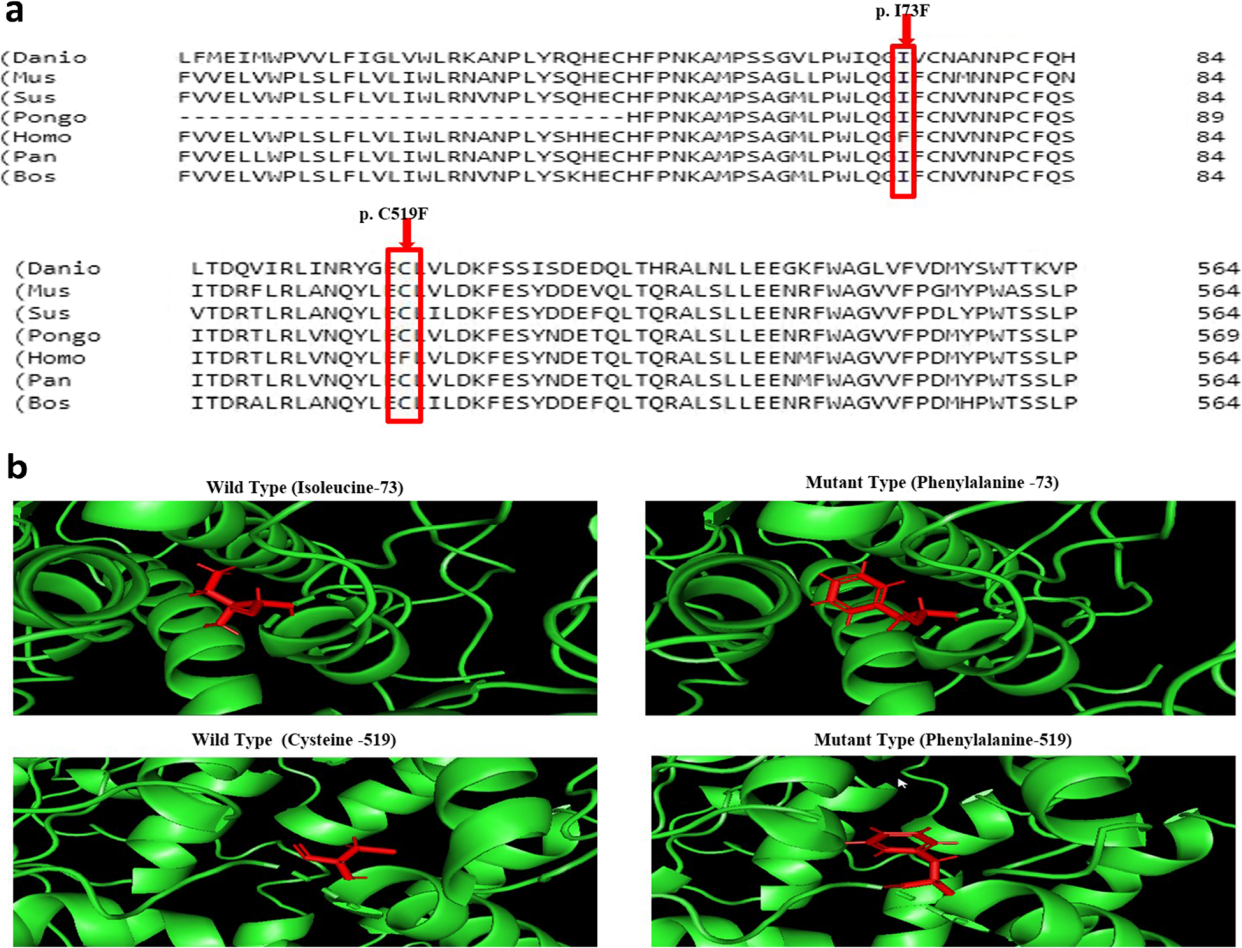
Conservation analysis and structure prediction of wild-type and novel mutant ABCA4 proteins. **a** Multiple sequence alignment of human ABCA4 proteins with six different species (*Danio rerio*, *Mus musculus*, *Sus scrofa*, *Pongo pygmaeus*, *Homo sapiens*, *Pan paniscus* and *Bos taurus*) for identifying novel mutants revealed an alteration in a highly conserved amino acid - isoleucine to phenylalanine in case ID 25 and cysteine to phenylalanine in case ID 10. **b** Wild type and mutant type ABCA4 protein were predicted using I-TASSER online tool and viewed by PyMol version 2.3

Multiple sequence alignment was performed for the two novel missense variants with six different species. The sequence was observed to be 100% similar for both residues (p.C519F; p.I73F) (Fig. 3a). Further, the structure of ABCA4 exo-cytoplasmic domain (ECD-1; position 43-646) was predicted using I-TASSER tool. The modeling templates were retrieved from LOMETS (LOcal MEta-Threading-Server), a protein data bank (PDB) model 5XJY chosen as a template for predicting protein stability. Protein stability was identified based on the change in amino acid in the conserved region of the ECD-1 domain. Server (mCSM, SDM and DUET) results demonstrated that the missense mutations were destabilizing the ECD-1 region which was further emphasized by a minus value in Gibbs free energy [22] (Table 3). Wild and mutant residues were viewed using PyMol version 2.3 (Fig. 3b).

**Table 3:** Prediction of protein stability changes due to missense variant in *ABCA4*

Case ID	c.DNA change	Amino acid change	Zygosity	mCSM (Kcal/mol)	SDM (Kcal/mol)	DUET (Kcal/mol)	Prediction
10	c.G1556 T	p.C519F	Homozygous	−1.125	−0.55	−1.133	Destabilizing
25	c.A217T	p.I73F	Homozygous	−1.141	−0.25	−1.095	Destabilizing

Gibbs free energy change: destabilizing (< 0 kcal/mol) and stabilizing (> 0 kcal/mol)

*mCSM* = mutation cutoff scanning matrix

## Discussion

The present study identified *ABCA4* mutations in a South Indian population with a clinical phenotype of STGD1 disease using a combination of Sanger sequencing and clinical exome sequencing. The rate of homozygous variants identified in the population using the abovementioned methods was 75% (21/28)*.* Due to the small sample size and allelic heterogeneity of *ABCA4* mutants, it was not possible to establish a correlation between genetic data and the clinical phenotypic features of STGD1-affected patients. Foremost, the sequence analysis revealed missense, nonsense and compound heterozygous mutations involved in the disease pathogenesis of STGD1. This study further contributes to understanding the spectrum of *ABCA4* mutations in South Indian patients with STGD1 disease.

Sanger sequencing, a cost-effective approach, was adopted for precise molecular diagnosis. However, despite its accuracy, seven inconclusive cases were observed. Two out of seven patients showed benign variants rs3112831 [35] (Case ID: 1), rs142673376 (Case ID: 16) and the remaining five patients (Case IDs: 3, 7, 12, 15, 23) were found negative for the disease-causing mutation in *ABCA4*. The unsolved cases and cases harboring benign variants may be related to the following factors: (i) the clinical overlap might lead to distinct genetics. Therefore, other STGD candidate genes (e.g., *ELOVL4, PROM1, CNGB3*) may play a role in disease progression, (ii) Mutations in deep intronic region of *ABCA4* could be a cause for the typical STGD phenotype.

Previous studies reported a common hypomorphic allele of the *ABCA4* gene explaining the missing heritability in autosomal recessive disorders [36, 37]. In our cases, a hypomorphic allele rs1801581 (c.G2828A, p.R943Q) was identified in 25% (7/28) of STGD1 subjects that is reported to have a global minor allele frequency (GMAF - 0.01538) in healthy population. In vitro assay demonstrated the pathogenicity of the variant (p.R943Q) that had a minimal effect on nucleotidase activity and on nucleotide binding affinity [38]. This variant could be pathogenic only in *trans* allele condition to moderate the disease severity in STGD1 cases (IDs: 5 & 14), who possessed a disease-causing heterozygous mutation. Similarly, disease risk modulating variant (rs1801359) [39] was associated with heterozygous mutation in case ID: 6; which might be responsible for the late onset of phenotype expression in STGD1.

Two missense mutations (p.C519F; p.I73F) in case ID: 10 and case ID: 25 were observed which was not previously reported in the population database. Multiple sequence alignment of human (*Homo sapiens*) ABCA4 protein and other species’ ABCA4 protein region revealed that cysteine and isoleucine are highly conserved in the mutated region across the genus, suggesting that the mutated region may play role in the structural stability of the ABCA4 protein. The ABCA4 protein consists of two transmembrane domains (TMD) and two nucleotide binding domains (NBD) arranged in non-identical tandem halves (TMD1-NBD1-TMD2-NBD2) which is separated by exo-cytoplasmic domains (ECDs) [10]. Both novel mutations occurred at one of the large exocytoplasmic domains-1 (ECD-1), which is involved in the substrate translocation process with their highly mobile hinge domains [40].

Several reports showed that the common disease causing variant (c.5882G > A; p.G1961E) frequency was high in different ethnic cohorts like Somalia [41], those of Italian ancestry [42] and the Indian population [12, 34]. Patients exhibiting this variant (homozygous and compound heterozygous) were clinically classified as moderate severity or late-onset disease phenotype [33]. However, in vitro studies revealed a severe dysfunction due to this missense variant [11]. In the current study, fundus imaging of the variant-associated patients (Case IDs: 19, 25) who were in the early onset of disease progression revealed a severity of stages III and IV disease category. Further, ERG indicated cone-rod dysfunction. Similarly, case ID: 13 harbored the p.G1961E homozygous variant, who had vision problems (BCVA-20/200 in BE) from 26 years of age (clinical images not available).

This study described two missense mutations p.G396C and p.A967V for the first time in association with STGD1 in a South Indian population. In addition, two more disease-causing variants (p.Y665Ter, p.T1277 M) were observed that was consistent with the previous reports in an Indian population [31, 33].

## Conclusions

In conclusion, the clinical and genetic perspective of 28 unrelated STGD-like phenotype patients of South Indian origin indicated the diverse variants in *ABCA4*. However, the identified allelic heterogeneity was inconsistent with an earlier report [12]. In addition, it creates a setback in correlating the phenotypic-genotypic relation. Sanger sequencing is considered as a gold standard method to identify monogenic Mendelian disorders. Hence, this method was used to determine the disease causative variants in the candidate gene *ABCA4* that is associated with STGD1. In order to widen our knowledge, high throughput sequencing approach such as targeted exome sequencing was adopted to understand the genetic heterogeneity in our STGD1 phenotype. Due to a small number of samples and lack of clinical data, we were not able to explore the distinct genetics of STGD phenotype.

The prevalence rate of STGD remains to be investigated in the Indian population. In addition, the frequency of *ABCA4* is poorly understood in our cohort. Therefore, this preliminary study contributes to the allelic diversity and mutation rate of *ABCA4* in a South Indian population.

## Supplementary information

**Additional file 1: Table S1.** List of non-pathogenic variants identified in STGD patients (ID: 25, 26, 27, 28) by Targeted exome sequencing. **Table S2.** Segregation analysis of 11 unrelated probands. Segregation analysis was performed for parents of 11 unrelated probands; ^ß^ Consanguinity in parents; ^*^ Consanguinity in previous generation; ^#^ Non consanguinity in parents; ^†^ Genetic analysis was performed for affected sibling.

## Data Availability

All data generated or analyzed during this study are included in this published article and its supplementary information files.

## Abbreviations

ABCA4: ATP Binding Cassette Subfamily A Member 4
AF: Autofluorescence
BCVA: Best corrected visual acuity
ERG: Electroretinography
MAF: Minor allele frequency
PCR: Polymerase chain reaction
PE: Phosphatidylethanolamine
SD-OCT: Spectral-domain optical coherence tomography
STGD1: Stargardt1
TES: Targeted exome sequencing

## References

[1] Stargardt K (1909). Uber familiare, progressive degeenration under makulagegend des augen. Albrecht von Graefes Arch Ophthalmol.

[2] Weleber RG (1994). Stargardt’s macular dystrophy. Arch Ophthalmol.

[3] Walia S, Fishman GA (2009). Natural history of phenotypic changes in Stargardt macular dystrophy. Ophthalmic Genet.

[4] Moloney JB, Mooney DJ, O’Connor MA (1983). Retinal function in Stargardt’s disease and fundus flavimaculatus. Am J Ophthalmol.

[5] Fishman GA, Farber M, Patel BS, Derlacki DJ (1987). Visual acuity loss in patients with Stargardt’s macular dystrophy. Ophthalmology..

[6] Webster AR, Héon E, Lotery AJ, Vandenburgh K, Casavant TL, Oh KT (2001). An analysis of allelic variation in the *ABCA4*. Invest Ophthalmol Vis Sci.

[7] Stone EM, Nichols BE, Kimura AE, Weingeist TA, Drack A, Sheffield VC (1994). Clinical features of a Stargardt-like dominant progressive macular dystrophy with genetic linkage to chromosome 6q. Arch Ophthalmol.

[8] Donoso LA, Edwards AO, Frost A, Vrabec T, Stone EM, Hageman GS (2001). Autosomal dominant Stargardt-like macular dystrophy. Surv Ophthalmol.

[9] Azarian SM, Travis GH (1997). The photoreceptor rim protein is an ABC transporter encoded by the gene for recessive Stargardt’s disease (ABCR). FEBS Lett.

[10] Tsybovsky Y, Orban T, Molday RS, Taylor D, Palczewski K (2013). Molecular organization and ATP-induced conformational changes of ABCA4, the photoreceptor-specific ABC transporter. Structure..

[11] Garces Fabian, Jiang Kailun, Molday Laurie L., Stöhr Heidi, Weber Bernhard H., Lyons Christopher J., Maberley David, Molday Robert S. (2018). Correlating the Expression and Functional Activity of ABCA4 Disease Variants With the Phenotype of Patients With Stargardt Disease. Investigative Opthalmology & Visual Science.

[12] Battu Rajani, Verma Anshuman, Hariharan Ramesh, Krishna Shuba, Kiran Ravi, Jacob Jemima, Ganapathy Aparna, Ramprasad Vedam L., Kumaramanickavel Govindasamy, Jeyabalan Nallathambi, Ghosh Arkasubhra (2015). Identification of Novel Mutations inABCA4Gene: Clinical and Genetic Analysis of Indian Patients with Stargardt Disease. BioMed Research International.

[13] Miller SA, Dykes DD, Polesky HF (1988). A simple salting out procedure for extracting DNA from human nucleated cells. Nucleic Acids Res.

[14] Kumar P, Henikoff S, Ng PC (2009). Predicting the effects of coding non-synonymous variants on protein function using the SIFT algorithm. Nat Protoc.

[15] Adzhubei I, Jordan DM, Sunyaev SR (2013). Predicting functional effect of human missense mutations using PolyPhen-2. Curr Protoc Hum Genet.

[16] Desmet FO, Hamroun D, Lalande M, Collod-Béroud G, Claustres M, Béroud C (2009). Human splicing finder: an online bioinformatics tool to predict splicing signals. Nucleic Acids Res.

[17] Schwarz JM, Rödelsperger C, Schuelke M, Seelow D (2010). MutationTaster evaluates disease-causing potential of sequence alterations. Nat Methods.

[18] Dong C, Wei P, Jian X, Gibbs R, Boerwinkle E, Wang K (2015). Comparison and integration of deleteriousness prediction methods for nonsynonymous SNVs in whole exome sequencing studies. Hum Mol Genet.

[19] Kumaran M, Subramanian U, Devarajan B (2019). Performance assessment of variant calling pipelines using human whole exome sequencing and simulated data. BMC Bioinformatics.

[20] Wang K, Li M, Hakonarson H (2010). ANNOVAR: functional annotation of genetic variants from high-throughput sequencing data. Nucleic Acids Res.

[21] Stelzer G, Plaschkes I, Oz-Levi D, Alkelai A, Olender T, Zimmerman S (2016). VarElect: the phenotype-based variation prioritizer of the GeneCards suite. BMC Genomics.

[22] Milenkovic Andrea, Milenkovic Vladimir M, Wetzel Christian H, Weber Bernhard H F (2018). BEST1 protein stability and degradation pathways differ between autosomal dominant Best disease and autosomal recessive bestrophinopathy accounting for the distinct retinal phenotypes. Human Molecular Genetics.

[23] Chun R, Fishman GA, Collison FT, Stone EM, Zernant J, Allikmets R (2014). The value of retinal imaging with infrared scanning laser ophthalmoscopy in patients with stargardt disease. Retina.

[24] Lois N, Holder GE, Bunce C, Fitzke FW, Bird AC (2001). Phenotypic subtypes of Stargardt macular dystrophy–fundus flavimaculatus. Arch Ophthalmol.

[25] Ernest PJ, Boon CJ, Klevering BJ, Hoefsloot LH, Hoyng CB (2009). Outcome of ABCA4 microarray screening in routine clinical practice. Mol Vis.

[26] Singh HP, Jalali S, Narayanan R, Kannabiran C (2009). Genetic analysis of Indian families with autosomal recessive retinitis pigmentosa by homozygosity screening. Invest Ophthalmol Vis Sci.

[27] Rivera A, White K, Stöhr H, Steiner K, Hemmrich N, Grimm T (2000). A comprehensive survey of sequence variation in the ABCA4 (ABCR) gene in Stargardt disease and age-related macular degeneration. Am J Hum Genet.

[28] Fujinami K, Zernant J, Chana RK, Wright GA, Tsunoda K, Ozawa Y (2013). ABCA4 gene screening by next-generation sequencing in a British cohort. Invest Ophthalmol Vis Sci.

[29] Allikmets R, Singh N, Sun H, Shroyer NF, Hutchinson A, Chidambaram A (1997). A photoreceptor cell-specific ATP-binding transporter gene (ABCR) is mutated in recessive Stargardt macular dystrophy. Nat Genet.

[30] Maugeri A, Klevering BJ, Rohrschneider K, Blankenagel A, Brunner HG, Deutman AF (2000). Mutations in the ABCA4 (ABCR) gene are the major cause of autosomal recessive cone-rod dystrophy. Am J Hum Genet.

[31] Zhang X, Ge X, Shi W, Huang P, Min Q, Li M (2014). Molecular diagnosis of putative Stargardt disease by capture next generation sequencing. PLoS One.

[32] Stenirri S, Fermo I, Battistella S, Galbiati S, Soriani N, Paroni R (2004). Denaturing HPLC profiling of the ABCA4 gene for reliable detection of allelic variations. Clin Chem.

[33] Burke TR, Fishman GA, Zernant J, Schubert C, Tsang SH, Smith RT (2012). Retinal phenotypes in patients homozygous for the G1961E mutation in the ABCA4 gene. Invest Ophthalmol Vis Sci.

[34] Lee W, Schuerch K, Zernant J, Collison FT, Bearelly S, Fishman GA (2017). Genotypic spectrum and phenotype correlations of ABCA4-associated disease in patients of south Asian descent. Eur J Hum Genet.

[35] López-Rubio S, Chacon-Camacho OF, Matsui R, Guadarrama-Vallejo D, Astiazarán MC, Zenteno JC. Retinal phenotypic characterization of patients with *ABCA4* retinopathy due to the homozygous p.Ala1773Val mutation. Mol Vis. 2018;24:105–14. PMC5800431 29422768

[36] Bauwens M, Garanto A, Sangermano R, Naessens S, Weisschuh N, De Zaeytijd J (2019). ABCA4-associated disease as a model for missing heritability in autosomal recessive disorders: novel noncoding splice, cis-regulatory, structural, and recurrent hypomorphic variants. Genet Med.

[37] Zernant J, Lee W, Collison FT, Fishman GA, Sergeev YV, Schuerch K (2017). Frequent hypomorphic alleles account for a significant fraction of ABCA4 disease and distinguish it from age-related macular degeneration. J Med Genet.

[38] Suárez T, Biswas SB, Biswas EE (2002). Biochemical defects in retina-specific human ATP binding cassette transporter nucleotide binding domain 1 mutants associated with macular degeneration. J Biol Chem.

[39] Schulz HL, Grassmann F, Kellner U, Spital G, Rüther K, Jägle H (2017). Mutation spectrum of the ABCA4 gene in 335 Stargardt disease patients from a multicenter German cohort-impact of selected deep intronic variants and common SNPs. Invest Ophthalmol Vis Sci.

[40] Pollock NL, McDevitt CA, Collins R, Niesten PH, Prince S, Kerr ID (2014). Improving the stability and function of purified ABCB1 and ABCA4: the influence of membrane lipids. Biochim Biophys Acta.

[41] Guymer RH, Héon E, Lotery AJ, Munier FL, Schorderet DF, Baird PN (2001). Variation of codons 1961 and 2177 of the Stargardt disease gene is not associated with age-related macular degeneration. Arch Ophthalmol.

[42] Passerini I, Sodi A, Giambene B, Mariottini A, Menchini U, Torricelli F (2010). Novel mutations in of the ABCR gene in Italian patients with Stargardt disease. Eye (Lond).

