# Supplementary material for: Genetic characterization of Stargardt clinical phenotype in South Indian patients using sanger and targeted sequencing
Source: Eye Vis (Lond). 2020 Jan 9;7:3. doi: 10.1186/s40662-019-0168-8 (PMC6950877; doi:10.1186/s40662-019-0168-8)
Supplement: Supplementary file 1 — Additional file 1: Table S1. List of non-pathogenic variants identified in STGD patients (ID: 25, 26, 27, 28) by Targeted exome sequencing. Table S2. Segregation analysis of 11 unrelated probands. Segregation analysis was performed for parents of 11 unrelated probands; ß Consanguinity in parents; * Consanguinity in previous generation; # Non consanguinity in parents; † Genetic analysis was performed for affected sibling. [file 40662_2019_168_MOESM1_ESM.docx]

**Additional files:**

**Table 1.** List of non-pathogenic variants identified in STGD patients (ID: 25, 26, 27, 28) by Targeted exome sequencing.

**Supplementary Table 1.** List of non-pathogenic variants identified in STGD patients (IDs: 25, 26, 27, 28) by clinical exome sequencing.

|  | **ID:25** | | **ID:26** | | **ID:27** | | **ID:28** | |
| --- | --- | --- | --- | --- | --- | --- | --- | --- |
| **Gene** | **Nucleotide**  **Exchange** | **SNP ID** | **Nucleotide**  **Exchange** | **SNP ID** | **Nucleotide**  **Exchange** | **SNP ID** | **Nucleotide**  **Exchange** | **SNP ID** |
| CNGB3 | c.892A>C | rs886063161 | c.919A>G | rs13265557 | c.892A>C | rs886063161 | c.892A>C | rs886063161 |
|  | c.702T>G | rs6471482 | c.892A>C | rs886063161 | c.702T>G | rs6471482 | c.702T>G | rs6471482 |
|  | - | - | c.702T>G | rs6471482 | c.494-11dup | rs36008065 | - | - |
| ELOVL4 | c.1795T>A | rs343705 | c.895A>G | rs3812153 | c.370-26A>T | - | c.895A>G | rs3812153 |
|  | c.895A>G | rs3812153 | c.370-26A>T | - | - | - | c.370-26A>T | - |
|  | c.370-26A>T | - | - | - | - | - | c.289-29T>C | - |
| PROM1 | c.2462+159dup | rs3841512 | c.2489+159dup | - | c.2489+159dup | - | c.2489+159dup | - |
|  | c.2462+118del | rs58036643 | c.2374-4dup | rs34269395 | c.2489+118del | - | c.2489+118dup | - |
|  | c.277-2220G>A | rs2078622 | c.1983+43T>C | - | c.2374-4dup | rs34269395 | c.303+6G>A | rs2078622 |
|  | c.-213+243T>C | rs7692760 | c.1983+14G>A | rs4698436 | c.1983+43T>C | - | - | - |
|  | - | - | c.303+6G>A | rs2078622 | c.1983+14G>A | rs4698436 | - | - |
|  | - | - | c.221-16191G>T | - | c.303+6G>A | rs2078622 | - | - |
|  | - | - | - | - | c.221-16191G>T | - | - | - |

**Table 2.** Segregation analysis of 11 unrelated probands.

**Supplementary Table 2.** ABCA4 genotypes in Stargardt disease patients.

| Case ID | Exon **/** Intron | Allele 1 | | Exon **/** Intron | Allele 2 | |
| --- | --- | --- | --- | --- | --- | --- |
|  |  | c.DNA change | Amino acid change |  | c.DNA change | Amino acid change |
| **^ß^**17 | 48 | c.C6658T | Q2220Ter | 48 | c.C6658T | Q2220Ter |
| **^*^**18 | 48 | c.C6658T | Q2220Ter | 48 | c.C6658T | Q2220Ter |
| **^#^**19 | 14 | c.C1995A | Y665Ter | 19,42 | c.C2912A , c.G5882A | T971A , G1961E |
| **^#^**20 | 46**†** | c.6355DelC | - | 48 | c.C6658T | Q2220Ter |
| **^#^**21 | 26 **/** 33**†** | c.C3830T **/** c.4774-2A>G | T1277M **/ -** | **-** | **-** | **-** |
| **^*^**22 | 44 | c.A6095G | H2032R | 44 | c.A6095G | H2032R |
| **^#^**24 | 19 | c.C2900T | A967V | 19 | c.C2900T | A967V |
| **^ß^**25 | 3 | c.A217T | I73F | 42 | c.G5882A | G1961E |
| **^ß^**26 | 19 | c.C2912A | T971A | 19 | c.C2912A | T971A |
| **^ß^**27 | 22 | c.G3323A | A1108H | 22 | c.G3323A | A1108H |
| **^ß^**28 | 19 | c.C2912A | T971A | 19 | c.C2912A | T971A |

Segregation analysis was performed for parents of 11 unrelated probands; ^ß^ Consanguinity in parents; ^*^ Consanguinity in previous generation; ^#^ Non consanguinity in parents; ^†^ Genetic analysis was performed for affected sibling.
